# Maternal Work and Spontaneous Preterm Birth: A Multicenter Observational Study in Brazil

**DOI:** 10.1038/s41598-020-66231-2

**Published:** 2020-06-16

**Authors:** Mariana Buen, Eliana Amaral, Renato T. Souza, Renato Passini, Giuliane J. Lajos, Ricardo P. Tedesco, Marcelo L. Nomura, Tábata Z. Dias, Patrícia M. Rehder, Maria Helena Sousa, José Guilherme Cecatti, the Brazilian Multicentre Study on Preterm Birth Study Group†, Sérgio T. Marba, Jacinta P. Matias, Nelson L. Maia Filho, Vera T. M. Borges, Laércio R. Oliveira, Tenilson A. Oliveira, Augusta M. B. Assumpção, Maria E. L. Moreira, Marcela Guedes, Cintia E Senger, Janete Vettorazzi, Francisco E. Martinez, Silvana M. Quintana, Patricia P. S. Melli, Antonio C. F. Barbosa Lima, Debora F. Leite, Elias Melo Junior, Denis J. Nascimento, Edson G. Tristão, Luciana F. Siqueira, Pedro R. Coutinho, Ruth Guinsburg, Ana C. P. Zamarian, Eduardo Souza, Marilia G. Martins, Marynea V. Nunes, Claudio S. M. Paiva, Djacyr M. C. Freire, Moisés D. Lima, Ana M. F. Porto, Isabela C. Coelho, Adriana S. O. Melo, Fabiana O. Melo, Melânia M. R. Amorim, Carlos A. S. Menezes, Marcelo Aquino, Francisco E. L. Feitosa, George N. Chaves, Miriam R. F. Silveira, Nelson Sass, Fátima A. H. Lotufo, Kaliane P. Uzilin, Carla B. Andreucci, Elvira A. O. Zanette

**Affiliations:** 1 Department of Obstetrics & Gynecology, University of Campinas (Unicamp), School of Medicine, São Paulo, Brazil; 2 Jundiai School of Medicine, Jundiai, SP Brazil; 3 Faculdade de Medicina de Botucatu - UNESP -, Botucatu, SP Brazil; 4 Casa Maternal Leonor Mendes de Barros -, São Paulo, SP Brazil; 5 FIOCRUZ - Instituto Fernandes Figueira -, Rio de Janeiro, RJ Brazil; 6 Hospital de Clinicas de Porto Alegre -, Porto Alegre, RS Brazil; 7 Hospital das Clinicas da FMRP-USP -, Ribeirão Preto, SP Brazil; 8 Hospital das Clínicas da UFPE -, Recife, PE Brazil; 9 Hospital das Clínicas da Universidade Federal do PR -, Curitiba, PR Brazil; 10 Hospital Estadual de Sumaré -, Sumaré, SP Brazil; 11 Hospital São Paulo - UNIFESP -, São Paulo, SP Brazil; 12 Hospital Universitário da UFMA -, São Luis, MA Brazil; 13 Hospital Universitário Lauro Wanderley da UFPB -, João Pessoa, PB Brazil; 14 IMIP Instituto de Medicina Integral Prof. Fernando Figueira -, Recife, PE Brazil; 15 Instituto de Saúde Elpídio de Almeida (ISEA) -, Campina Grande, PB Brazil; 16 Maternidade Climério de Oliveira -, Salvador, BA Brazil; 17 Maternidade Escola Assis Chateaubriand -, Fortaleza, CE Brazil; 18 Maternidade Escola de Vila Nova Cachoeirinha Dr. Mario Moraes A. Silva -, São Paulo, SP Brazil; 19 Santa Casa de Limeira -, Limeira, SP Brazil; 20 Santa Casa de São Carlos -, São Carlos, SP Brazil

**Keywords:** Medical research, Epidemiology

## Abstract

Spontaneous preterm birth (sPTB) is a major pregnancy complication involving biological, social, behavioural and environmental mechanisms. Workload, shift and intensity may play a role in the occurrence of sPTB. This analysis is aimed addressing the effect of occupational activities on the risk for sPTB and the related outcomes. We conducted a secondary analysis of the EMIP study, a Brazilian multicentre cross-sectional study. For this analysis, we included 1,280 singleton sPTB and 1,136 singleton term birth cases. Independent variables included sociodemographic characteristics, clinical complications, work characteristics, and physical effort devoted to household chores. A backward multiple logistic regression analysis was applied for a model using work characteristics, controlled by cluster sampling design. On bivariate analysis, discontinuing work during pregnancy and working until the 7^th^ month of pregnancy were risks for premature birth while working during the 8^th^ - 9^th^ month of pregnancy, prolonged standing during work and doing household chores appeared to be protective against sPTB during pregnancy. Previous preterm birth, polyhydramnios, vaginal bleeding, stopping work during pregnancy, or working until the 7^th^ month of pregnancy were risk factors in the multivariate analysis. The protective effect of variables compatible with exertion during paid work may represent a reverse causality. Nevertheless, a reduced risk associated with household duties, and working until the 8^th^-9^th^ month of pregnancy support the hypothesis that some sort of physical exertion may provide actual protection against sPTB.

## Introduction

Preterm birth (PTB) remains a co mplex health problem. Risks for PTB vary according to ethnicity, geography and factors related to lifestyle^1^. Recent studies have shown that preterm complications cause 29% of neonatal deaths and are responsible for significant morbidity after birth^2,3^. Prematurity may exert a negative impact due to sequelae of the newborn, loss of the fetus, emotional distress of the family and enormous medical costs associated with the use of different therapeutic resources. As a result of technological advances and improved medical care, many preterm infants survive with less disability. However, these children may remain vulnerable to long-term complications which can last a lifetime^4^.

In 75% of cases, preterm birth is spontaneous and has a multifactorial origin. It appears that risk factors vary according to gestational age, as well as social and environmental aspects^5^. Socioeconomic and educational levels may increase or decrease the risks of sPTB, interacting with other factors. In California, the odds of having PTB were greater in white women with lower socioeconomic conditions than in black women. In white families living at the poverty level, the prematurity rate was 10.4%, decreasing to 6% in upper-income families. Among black families, prematurity rates were 12.5 and 15.8%, respectively. The decreasing trend observed was dependent on the level of maternal schooling. White women with lower levels of school education (up to high school) had 12.7% of premature deliveries, compared to 5.9% in more highly educated women (up to college or beyond)^6^. Nevertheless, data from the Brazilian Demographic Health Survey (DHS) showed that white women, women from urban areas, history of hypertension or heart disease, twin gestations, non-elective Caesarean sections, health insurance for delivery, low number of antenatal visits, and severe morbidity were all maternal factors associated with preterm birth^7^.

Among the potential risk factors for PTB, maternal exertion during paid work was also addressed. Previous studies have found that paid work increased the risk for PTB^8–10^. Women whose job required prolonged standing had a moderately increased risk of sPTB^8–11^, since a static and standing position could damage primarily uterine blood flow. There was also the risk of sPTB in pregnant women working long hours maintaining the same body position (more than six hours, either sitting or standing)^12^, a job that required sitting and standing multiple times, shift work^10^, or working 40 hours or more per week^13^.

This association may vary according to the occupational activity. In a study carried out in Europe, the employed group during pregnancy had less risk of PTB. This group was composed of nurses, part of the chief female occupational sector. In contrast, food industry workers and unemployed women, including students and stay-at-home women had an increased risk of preterm delivery^14^.

According to the Brazilian Institute of Geography and Statistics (IBGE) data, in the first trimester of 2015, women accounted for 52.3% of the population over 14 years of age working in the most heavily populated regions of the country^15^. Thus, we believe that a study of the potential risk factors for preterm birth associated with paid work seems opportune.

The EMIP study (Brazilian Multicenter Study on Preterm Birth) was developed as a cross-sectional study evaluating all preterm births in 20 referral obstetric hospitals in the northeast, south and southeast of the country, associated with a nested case-control component. Spontaneous preterm births (cases) were then compared with full-term births (controls) to identify and analyze the main risk factors associated with preterm births^16^. The current analysis is aimed at evaluating maternal exertion during paid work associated with spontaneous preterm birth among women from the EMIP study.

## Methods

### Study design

**Figure 1:**
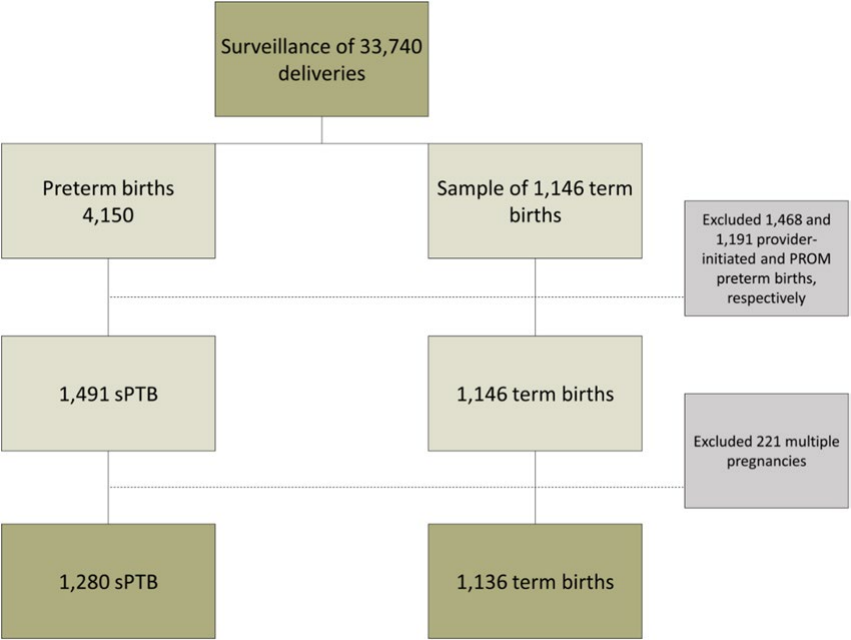
Flowchart of the EMIP study: singleton spontaneous preterm (sPTB) and term birth cases.

This is a secondary analysis from a multicenter cross-sectional study with a nested case-control component that addresses the prevalence, risk factors and related perinatal outcomes for preterm births in Brazil. We are currently focused on the association between maternal work characteristics and sPTB among singleton pregnancies. Figure 1 shows the flowchart of the EMIP study: the control group was nested to the cross-section component. All births in the participating centers were surveilled; all women who had preterm birth were invited to participate (cross-sectional component). Then, a control group composed of a sample of women who had term birth were invited to participate and they were compared to the preterm birth group (case-control component).

### Population and sample size

The EMIP study carried out the surveillance of 33,740 deliveries in 20 Brazilian referral obstetric centres in three regions (South, Southeast, and Northeast), resulting in 4,150 preterm births (<37 weeks) included from April 2011 to July 2012. Sample size calculation was based on a prevalence of 6.5% of preterm births in Brazil, according to national vital statistics for 2011 and an estimated odds ratio of 1.4 for smoking. Type I error was 0.05, type II error was 0.10 (β), power of 90% (1-β) and losses were estimated at 4%^16^. According to the sample size calculation, 1,054 women would be necessary in each group (spontaneous onset of preterm labor, preterm premature rupture of membranes, provider-initiated PTB and term births (control) groups). Women with term birth that occurred immediately after that specific case of preterm birth were invited to participate and composed the control group. Considering that the frequency of term birth was much higher than preterm births, women with term birth were invited to participate until the sample size was reached for this group. The study dataset included a control group comprised of 1,146 women who had a term delivery (≥37 weeks). Of the women with preterm births, 1,468 were provider-initiated, when it was medically indicated due to fetal and/or maternal conditions, 1,491 were sPTB, due to spontaneous onset of labor, and 1,191 were PROM-PTB, due to premature rupture of membranes. For this analysis, women who had PROM-PTB, provider-initiated PTB and twins were excluded (Fig. 1).

### Variables

Independent variables related to duration and exertion at the workplace and while doing household chores, derived from the EMIP data set, were analysed. These dichotomous variables included: until which month of pregnancy the woman had worked (classified as not working or up to the 7^th^ month, and the 8–9^th^ month), intensity of effort at the workplace during pregnancy (classified as without work or not intense work, and sometimes or always intense work), standing position during work (classified as without work or not working in the standing position, and working sometimes or predominantly on their feet during pregnancy), working hours during pregnancy (classified as without work or working up to 8 hours per day, and working more than 8 hours), working the night shift (classified as without work, not working, or sometimes working the night shift, and working the night shift) and household chores (classified as not working at home or working at home with help, and working at home unaided). Other variables such as socioeconomic, demographic, personal and obstetric history were considered for risk factors. These included age, living without a partner, schooling, initial body mass index (BMI), total weight gain during pregnancy, drug addiction before and during pregnancy, smoking, bleeding during pregnancy, previous preterm births, history of hospital admission due to preterm birth, history of PROM, previous C-section, and maternal morbidities such as anemia and hypertension.

Women were classified according to work characteristics including status, period and intensity of work. Work status was defined as never worked (during the index pregnancy), unpaid work (informal job or household chores), and paid work. Work period included never worked, worked until the 7^th^ month of pregnancy, worked until the 9^th^ month of pregnancy. Work intensity was categorized as not intense and intense. Intense work was defined as having any of the following: working the night shift, more than 8 h per day, standing required, or intense physical effort.

### Data collection

Data collection from the EMIP study was carried out as follows. Postpartum women were invited and signed an Informed Consent Form after agreeing to participate in the study. Later, an interview was conducted and data was collected using a specific data collection tool (questionnaire) dedicated to this study. The questionnaire had two parts. The first part included patient identification, socioeconomic data, obstetric history, previous morbidity, conditions during this pregnancy, multiple gestations, conditions related to PTB listed above, data on delivery and newborn, including neonatal morbidity and mortality. The second part was completed only when there was a premature delivery, informing whether it had been spontaneous, therapeutic or elective, and if PROM had occurred^16,17^. OpenClinica version 3.0 (http://openclinica.org/) was the software used for electronic capture of EMIP data^18^, by a trained study coordinator and supervisor revising the data entered in each hospital, and later at the central level. Details on data collection are available from previously published papers^16,17^.

### Statistical analysis

After a descriptive analysis to define the sample for this approach, a bivariate analysis was performed to estimate the risks of some sociodemographic, behavioral, clinical and obstetrical characteristics with spontaneous preterm births (sPTB), providing OR and their respective 95% confidence intervals. The same approach was then performed for maternal work characteristics and spontaneous preterm birth. Then, a backward multiple analysis by non-conditional logistic regression was applied to identify all characteristics independently associated with spontaneous PTB, considering those individual variables with p < 0.20. Finally, perinatal outcomes for all preterm births were compared according to maternal work groups using χ^2^ tests. All analyses were controlled by cluster sampling design. The units at the first level of sampling were called Primary Sampling Units (PSU); in this study, participating obstetric centers (maternities) were the PSUs. Because of the sampling design, observations in the same cluster are not independent and the variance estimators allow any amount of correlation within the PSU and they produce variance estimates that generally will be either approximately unbiased or biased toward more conservative estimates (larger standard errors). Intracluster correlation coefficients were considered low (close to zero) in most variables of the EMIP study, showing intracluster heterogeneity. All p-values, odds ratios and respective confidence intervals accounted for the cluster sampling design. The statistical significance level was set at a p-value < 0.05. For data analysis, the SPSS program, version 20.0^19^ and the Stata program, version 7.0^20^ were used. This manuscript follows the STROBE statement^21^.

### Ethical aspects

Ethical approval for the research protocol was obtained from the National Council for Ethics in Research (CONEP)^16^. Also, the Institutional Review Boards of the following institutions reviewed and approved this study: Maternidade Climério de Oliveira (Salvador, BA), Maternidade Escola Assis Chateaubriand (Fortaleza, CE), Hospital Universitário da Universidade Federal do Maranhão (Sao Luis, MA), Instituto de Saúde Elidio de Almeida (Campina Grande, PB), Hospital Universitário Lauro Wanderley da Universidade Federal da Paraíba (Joao Pessoa, PB), Instituto de Medicina Integral Prof. Fernando Figueira (Recife, PE), Hospital das Clinicas da Universidade Federal de Pernambuco (Recife, PE), Hospital das Clinicas da Universidade Federal do Paraná (Curitiba, PR), Instituto Fernandes Figueira (Rio de Janeiro, RJ), Hospital das Clinicas da Universidade Federal do Rio Grande do Sul (Porto Alegre, RS), Faculdade de Medicina de Botucatu da Universidade Estadual Paulista (Botucatu, SP), Hospital da Mulher da Universidade Estadual de Campinas (Campinas, SP), Maternidade Escola de Vila Nova Cachoeirinha (São Paulo, SP), Hospital Estadual de Sumaré (Sumaré, SP), Faculdade de Medicina de Jundiaí (Jundiaí, SP), Hospital das Clinicas da Faculdade de Medicina de Ribeirão Preto da Universidade de São Paulo (Ribeirão Preto, SP), Santa Casa de Limeira (Limeira, SP), Santa Casa de São Carlos (São Carlos, SP), Casa Maternal Leonor Mendes de Barros (São Paulo, SP), and Hospital São Paulo da Universidade Federal de São Paulo (São Paulo, SP).

Before entering the study, all women read, agreed and signed an Informed Consent Form. All methods were performed in accordance with the Brazilian National Health Council (Resolution CNS 466/12), conforming to local regulations in all steps of the study. To guarantee the quality of data collected, some procedures were performed, such as initial training meetings for research assistants and local investigators, development of standard operating procedures (SOP) for management of the questionnaire and database, monitoring site visits, sustained online monitoring of data entry and correctness by the coordinating center staff.

## Results

The EMIP study included 4,150 women with preterm births and 1,146 with term births. After excluding PROM and provider-initiated preterm births and multiple pregnancies, our analysis considered 1,280 women with sPTB and 1,136 with term births. The methods for estimating gestational age at delivery (LMP, US and New Ballard) did not statistically differ between sPTB and term groups (Suppl Info; Table S1). Table 1 shows the maternal characteristics of women who had sPTB and term births. Women who had sPTB were more likely at both extremes of the reproductive age, had no partner, had a lower schooling level and were unemployed. Despite statistical differences in characteristics between groups, there were no remarkable differences. On bivariate analysis, risk factors for spontaneous preterm birth were: age under 20 years, drug addiction before and during pregnancy, bleeding during pregnancy, previous preterm births, weight gain during pregnancy ≤10 kg, BMI < 20, ≤ 8 years of schooling, smoking >10 cigarettes per day, living without a partner, and history of premature rupture of membranes. In contrast, women who had previous cesarean sections, any maternal chronic morbid condition, presence of arterial hypertension, preeclampsia/eclampsia/HELLP syndrome, in addition to household chores were less likely to have preterm birth (Table 2).

**Table 1:** Sociodemographic characteristics of the study population.

Maternal characteristics	sPTB N (%)	Term birth N (%)	p-value
**Maternal age (years)**			**<0.001**
≤ 19	399 (31.2)	211 (18.6)	
20–34	767 (59.9)	800 (70.4)	
≥ 35	114 (8.9)	125 (11.0)	
**Skin colour/ethnicity**			0.273
White	552 (43.1)	447 (39.3)	
Other	728 (56.9)	689 (60.7)	
**Marital status**			**0.009**
With a partner	961 (75.1)	912 (80.3)	
Without a partner	319 (24.9)	224 (19.7)	
**Household** (a)			0.953
Urban	1140 (89.6)	1012 (89.5)	
Rural	133 (10.4)	119 (10.5)	
**Schooling (years) (b)**			**0.016**
≤8	562 (44.6)	419 (37.4)	
9–12	628 (49.8)	622 (55.5)	
>12	71 (5.6)	79 (7.1)	
**Paid work in pregnancy** (c)			**<0.001**
No	884 (69.4)	684 (60.3)	
Yes	389 (30.6)	451 (39.7)	
**Workload (daily)** [n = ] (d)			0.170
≤ 8 hours	1097 (90.7)	965 (88.4)	
> 8 hours	112 (9.3)	127 (11.6)	
**Children under 5 years** (e)			0.069
No	883 (69.0)	814 (71.7)	
Yes	397 (31.0)	321 (28.3)	
**Total**	**1280 (100)**	**1136 (100)**	

Values in bold mean they are statistically significant.

Missing information for: (a): 12; (b): 35; (c): 8; (d): 115; (e): 1.

**Table 2:** Estimated risks of some sociodemographic, behavioral, clinical and obstetrical characteristics with spontaneous preterm births (sPTB) in the EMIP study.

Characteristics	sPTB N (%)	Term birth N (%)	OR	95%CI
Age <20 years	399 (31.2)	211 (18.6)	**1.99**	**[1.71–2.31]**
Age ≥ 35 years	114 (8.9)	125 (11.0)	0.79	[0.57–1.09]
Without a partner	319 (24.9)	224 (19.7)	**1.35**	**[1.09–1,68]**
School education ≤ 8 years (a)	562 (44.6)	419 (37.4)	**1.35**	**[1.08–1.67]**
Weight gain ≤ 10 kg (b)	689 (63.4)	396 (39.3)	**2.67**	**[2.19–3.26]**
Previous PTB (c)	140 (11.0)	62 (5.5)	**2.14**	**[1.44–3.17]**
Drug addiction before/during	88 (6.9)	41 (3.6)	**1.97**	**[1.32–2.94]**
Bleeding during Pregnancy (d)	353 (27.6)	185 (16.3)	**1.96**	**[1.48–2.61]**
Polyhydramnios (e)	35 (3.1)	17 (1.7)	1.88	[0.97–3.64]
BMI < 25 kg/m^2^ (f)	304 (28.0)	178 (17.8)	**1.80**	**[1.43–2.27]**
Smoking >10 cigarettes/day	79 (6.2)	39 (3.4)	**1.85**	**[1.33–2.57]**
Smoking until 9 months pregnant	219 (17.1)	123 (10.8)	**1.70**	**[1.30–2.22]**
Smoking ≥ 1 cigarette/day	218 (17.0)	123 (10.8)	**1.69**	**[1.30–2.20]**
History of preterm PROM (g)	117 (9.2)	64 (5.7)	**1.69**	**[1.17–2.44]**
No previous child or aged <1 year (h)	719 (56.2)	568 (50.0)	**1.28**	**[1.02–1.61]**
Lives with a smoker (i)	433 (34.2)	350 (30.9)	1.16	[0.98–1.38]
Children aged <5 years (h)	397 (31.0)	321 (28.3)	1.14	[0.99–1.31]
Previous cesarean sections (h)	220 (17.2)	247 (21.7)	**0.75**	**[0.58–0.97]**
Any chronic morbid condition (j)	264 (21.8)	318 (29.7)	**0.66**	**[0.44–0.99]**
Chronic hypertension (j)	22 (1.8)	30 (2.8)	0.64	[0.37–1.12]
Preeclampsia/Eclampsia/HELLP (j)	**52 (4.3)**	**90 (8.4)**	**0.49**	**[0.29–0.83]**
Total	1280	1136		

Non-associated variables (p < 0.10): residential zone, skin color, family income, paved street, piped water, sewage system, alcohol consumption, treatment of vaginal discharge, treatment of urinary tract infection, anemia during the current pregnancy, number of pregnancies, intense physical exertion during pregnancy – frequent.

Missing information a) 35; b) 322; c) 7; d) 4; e) 243; f) 330; g) 9; h) 1; i) 15; j) 134.

Not working during pregnancy and working until the 7th month of pregnancy were associated with higher odds of having spontaneous preterm delivery, in comparison to never working. In contrast, women who worked until the 8^th^-9^th^ month of pregnancy, had prolonged standing during working hours, and domestic chores were less likely to have spontaneous preterm delivery (Table 3).

**Table 3:** Estimated risks of some work characteristics with sPTB in the EMIP study.

Characteristics of paid work	sPTB	Term birth	OR	95% CI
	N (%)	N (%)		
**Period of pregnancy when women worked (a)**				
Never worked	825 (64.8)	644 (56.7)	Ref.	
Without paid work during pregnancy or worked until 7th month	298 (23.4)	165 (14.5)	**1.41**	**[1.16–1.72]**
Worked until 8–9th month	150 (11.8)	326 (28.7)	**0.36**	**[0.27–0.48]**
**Intense physical effort (b)**				
Without work/No intense work	1,043 (86.1)	899 (82.2)	Ref.	
Yes/Sometimes	169 (13.9)	195 (17.8)	0.75	[0.55–1.02]
**Standing required (c)**				
Without work/No or Sometimes	1,004 (82.8)	849 (77.7)	Ref.	
Yes	208 (17.2)	244 (22.3)	**0.72**	**[0.59–0.88]**
**Working hours (d)**				
Without work/Up to 8 hours/day	1,097 (90.7)	965 (88.4)	Ref.	
More than 8 hours/day	112 (9.3)	127 (11.6)	0.78	[0.53–1.13]
**Working the night shift (e)**				
Without work/No/Sometimes	1,150 (95.3)	1,014 (92.9)	Ref..	
Yes	57 (4.7)	77 (7.1)	0.65	[0.42–1.01]
**Household work (f)**				
Not or Yes, aided	660 (51.6)	514 (45.3)	Ref.	
Yes, unaided	620 (48.4)	621 (54.7)	**0.78**	**[0.64–0.94]**
**Level of work (g)**				
No work or not intense	1,024 (85.3)	888 (81.8)	Ref.	
Intense	176 (14.7)	198 (18.2)	0.77	[0.59–1.00]

Missing information for a) 8; b) 110; c) 111; d) 115; e) 118; f) 1; g) 130.

Level of work defined as having two or more of the following: work with intense physical effort; standing; more than 8 h/day; at night.

On multiple logistic regression, factors independently associated with spontaneous preterm birth included previous PTB (OR_adj_ 3.88 [2.53–5.97]), polyhydramnios (OR_adj_ 3.78 [1.92–7.44]), vaginal bleeding (OR_adj_ 1.75 [1.22–2.51]), and no paid work/working until the 7th month (OR_adj_ 1.69 [1.27–2.24]), while protective factors against spontaneous preterm birth were age (OR_adj_ 0.97 [0.95–0.98]), BMI (OR_adj_ 0.92 [0.893–0.94]), weight gain (OR_adj_ 0.91 [0.89–0.93]), domestic chores unaided, anemia, and working until the 8^th^-9^th^ month of pregnancy (Table 4). Table 5 demonstrates perinatal outcomes according to the intensity and period of work during pregnancy. Women who worked until the 8–9^th^ month of pregnancy had a lower proportion of extremely and moderately preterm newborns compared to women with unpaid work or who worked until the 7^th^ month of pregnancy. Infants whose mothers worked until the end of pregnancy, therefore had less NICU admissions, neonatal deaths before discharge or any adverse perinatal outcome. Different levels of work intensity during pregnancy were not associated with adverse perinatal outcomes.

**Table 4:** Multiple logistic regression analysis of variables independently associated with spontaneous preterm birth among singleton pregnancies in the EMIP study (n = 1,756).

Variables	OR	95% CI	p-value
Weight gain during pregnancy (kg)	0.91	0.89–0.93	<0.001
Previous preterm delivery	3.93	2.60–5.95	<0.001
Age (<20 years)	1.71	1.37–2.14	<0.001
BMI < 25 kg/m^2^	2.14	1.56–2.94	<0.001
Worked up to 8–9th month	0.50	0.37–0.67	<0.001
Worked up to 7^th^ month	1.75	1.30–2.35	<0.002
Polihydramnios	3.71	1.89–7.27	<0.002
Vaginal bleeding during pregnancy	1.75	1.24–2.47	0.003
Anemia during pregnancy	0.74	0.62–0.89	0.003
Household work unaided	0.77	0.65–0.91	0.004
Antenatal care in primary health care unit	1.47	1.04–2.06	0.029

OR: Odds ratio; 95% CI: 95% Confidence interval for OR; p: p-value. Predictive variables considered in the model: Household chores performed in one’s household (Yes, unaided = 1/Yes, aided or No = 0); Paid work1 (Up to 7th month = 1); Paid work2 (Up to 8–9th month = 1); Standing during work (Yes/eventually = 1 or No = 0); Number of working hours (more than 8 h = 1 or up to 8 h = 0); Night shifts (Yes = 1 or Eventually/no = 0); Age (<20 years = 1); Marital status (With a partner = 0 or Without a partner = 1); School education (Up to 8 years = 0 or > 8 years = 1); Family income (Up to R$ 1.000,00 = 1 or > R$ 1.000,00 = 0); Chronic or complicating maternal morbidity (Yes, any condition one = 1 or No = 0); Lives where there is piped water (Yes = 1 or No = 0); Number of children aged less than 5 years (≥1 = 1 or None = 0); Age of youngest child (≥2 years = 1 or No other child/up to 1 year old = 0); BMI at the beginning of pregnancy (<25: 1/≥25 kg/m2:0); Weight gain during pregnancy (kg); Smoked during pregnancy (Yes = 1 or No = 0); Lives with a smoker (Yes = 1 or No = 0); Treatment of urinary tract infection during pregnancy (Yes = 1 or No = 0); Anemia during current pregnancy (Yes = 1 or No = 0); Vaginal bleeding during pregnancy (Yes = 1 or No = 0); Polyhydramnios (Yes = 1 or No = 0); Number of previous caesarean sections (≥1 = 1 or None = 0); Number of previous preterm births (Any = 1 or None = 0); History of hospital admission due to preterm labor (Yes = 1 or No = 0); History of rupture of membranes (Yes = 1 or No: 0); Prenatal care (Primary health care unit=1/ Other = 0).

**Table 5:** Perinatal outcomes according to maternal intensity of work and period of work during pregnancy in preterm births.

Perinatal outcomes	Intensity of work			Period of work			
	Never worked and Not intense work	Intense Work	p-value	Perinatal outcomes	Without paid work or until the 7^th^ month	Worked until the 8–9^th^ month	p-value
**Gestational age** [n = 1212]			0.529	**Gestational age** [n = 1273]			**<0.001**
**<28 weeks**	85 (8.1)	16 (9.5)		**<28 weeks**	111 (9.9)	—	
**28–33 weeks**	274 (26.3)	49 (29.0)		**28–33 weeks**	311 (27.7)	21 (14.0)	
**34–36 weeks**	684 (65.6)	104 (61.5)		**34–36 weeks**	701 (62.4)	129 (86.0)	
**Apgar score** < **7 at 5 minutes** [n = 1180]	115 (11.3)	18 (10.9)	0.916	**Apgar score** < **7 at 5 minutes** [n = 1238]	130 (11.9)	11 (7.6)	0.068
**NICU admission**[n = 1044]	495 (54.8)	81 (57.4)	0.627	**NICU admission** [n = 1097]	554 (56.8)	50 (41.0)	**0.007**
**Fetal death** [n = 1212]	31 (3.0)	8 (4.7)	0.127	**Fetal death** [n = 1273]	35 (3.1)	6 (4.0)	0.526
**Neonatal death before discharge** [n = 1170]	103 (10.2)	15 (9.3)	0.782	**Neonatal death before discharge** [n = 1229]	120 (11.1)	4 (2.8)	**0.003**
Any adverse perinatal outcome (APO)* [n = 1069]	543 (59.0)	91 (61.1)	0.698	Any adverse perinatal outcome (APO)* [n = 1122]	605 (60.8)	59 (46.5)	**0.012**

^*^Any adverse perinatal outcome (APO): Apgar score <7 at 5 minutes **or** NICU admission **or** neonatal death before discharge.

P-values in bold mean they are statistically significant.

## Discussion

This study showed that women who exerted themselves during work and completed household chores unaided were less likely to have spontaneous preterm births. Other factors with similar associations were age, high BMI, weight gain during pregnancy and anemia during pregnancy. In contrast, factors associated with an increased risk of spontaneous preterm birth were previous preterm deliveries, polyhydramnios and vaginal bleeding.

The unadjusted results should be interpreted with caution. Women who had previous cesarean sections, any maternal chronic morbid condition, presence of arterial hypertension, preeclampsia/eclampsia/HELLP syndrome, in addition to household chores were less likely to have preterm birth according to unadjusted estimative of risks. Possibly, this is a reflection of the phenotype of women composing the different comparison groups (women who had spontaneous preterm birth vs term birth). The control group (term birth) is comprised of women whose delivery occurred due to spontaneous onset of labor, PROM and provider-initiated (medically-indicated). Not only gestational age at birth (<37weeks and ≥37weeks) but also the clinical difference between the phenotypes should be accounted in the interpretation and clinical judgment of the findings.

A recent systematic review of the impact of occupational shift work and working hours on health outcomes included 59 studies in the meta-analysis^22^. The study showed that working rotating shifts, night shifts and working longer hours standing were associated with a higher risk for preterm delivery (OR 1.13, 95% CI [1.00–1.28], I^2^ = 31%; OR 1.21, 95% CI[1.03–1.42], I^2^ = 36%; OR 1.21, 95% CI [1.11–1.33], I^2^ = 30%, respectively). This association, however, is based on low or very low-quality evidence and the meta-analysis failed to take into account several confounders, such as socioeconomic status or recall bias. Our results differed from those of the literature. Some reasons may explain how paid work provided protection against preterm birth. It was probable that reverse causality occurred. Women who were at a higher risk for preterm delivery may have been instructed to decrease activities in the last trimester of pregnancy, despite continuing to work. It is possible that risk factors associated with work characteristics were identified and pregnant women at higher risk of PTB were granted temporary medical leave. Unfortunately, data analyzing the variation in job activities throughout pregnancy were not collected. In a Scandinavian study, aimed at investigating the association between work-related factors (posture, heavy lifting, shift work, working hours and stress at work) and risks of diseases during pregnancy and sick leave due to work-related disorders, it was observed that improved work conditions could prevent sick leave^23^. This finding is in agreement with changes in activities, resulting in potential risk reduction.

Another possibility is that work confers a real protective effect, associated with participation in moderate physical activity. Physical activity (PA) is considered any bodily movement produced by skeletal muscles, increasing calorie expenditure above levels at rest. Many studies confirm that physical activity is beneficial for pregnancy and exercise is currently recommended for the prevention of complications such as diabetes and hypertension^24,25^. According to the American College of Obstetricians and Gynecologists (ACOG), at least 30 minutes of moderate-intensity physical activity, at least 150 min/week, is recommended when there is no medical contraindication^26^.

Maternal exertion while doing household chores may have been greater than exertion at the workplace, generating such results. In the model of work characteristics, findings showed that working until the end of pregnancy and completing domestic chores unaided were protective factors. This finding reinforces the hypothesis that regular physical activity, in the form of paid jobs and household chores may best explain the decreased risk of preterm births.

Our findings are similar to those observed in a European study comparing pregnant women from various countries (Germany, Portugal, Italy, Switzerland, Denmark, Spain, Lithuania, Norway, France, Greece and Poland). In that study, pregnant women were classified as “non-working” when they had no formal profession (housewives, students, unemployed, and sick leave). The term “working” was used when these women had a formal job. Working women had a lower risk of preterm delivery when compared to the non-working group. Pregnant women who were not exposed to workplace hazards (chemical and physical agents), such as managers, teachers, administrators, had a lower risk of PTB than “exposed” pregnant women^14^.

In the late ‘80 s and early ‘90 s, studies focused mainly on occupational activities, and not on physical activity per se, especially leisure-time physical activity. During that time period, some authors found that certain occupational activities could be harmful during pregnancy. Among the characteristics of work activities, orthostatic posture (prolonged standing), long working hours and fatigue scores were studied. Other characteristics in the study included heavy lifting, walking up many flights of stairs and working constantly inclined in the standing or sitting posture^8,12,13,27–30^. However, none of those activities fit the definition of physical exercise, although they can be characterized as physical activity engaged during work duties or while doing household chores. The literature suggests that there is a combination of factors related to work/employment and a (lower or higher) risk of pregnancy complications such as preterm birth. The conception of the allostatic load is an attempt to understand and measure the burden of accumulated stress in the organism^31^. An interaction of diverse factors may contribute to coping with stressful situations related to work such as level of satisfaction with salary, stability, security, stress load (allostatic load), resiliency, etc. Evaluation of the effects of work intensity and work journey on the risk for sPTB is highly complex.

The effect of work conditions varies in the literature. The risk for post-term deliveries was increased in women with fixed night work. Fixed evening work was associated with term low birth weight, and shift work slightly increased small-for-gestational-age babies^32^. Among nurses, a part-time job reduced the risk for preterm birth and working at night tripled the risk for early preterm birth, at less than 32 weeks of gestation^33^. A previous metanalysis showed that physically demanding work was significantly associated with preterm birth, including prolonged standing, shift and night work, but not long hours, with an odds ratio smaller than 2.00 for identified risk factors^34^. A more recent review concluded that shift work, or night shifts during pregnancy was not significantly associated with an increased risk for PTB, highlighting the need for high-quality studies focused on risks per trimester^35^. There is a hypothetical relationship between daily rhythm patterns of the light/dark cycle (photoperiod) and mechanisms that determine parturition. Neuromodulation involving melatonin and other immunomodulators from both the mother and fetus throughout pregnancy and during the onset of labor may play a role in controlling parturition time^36^.

Research evaluating leisure-time physical activities (PA) emerged after the 1990s, either alone or in combination with other types of PA. Recent evidence has demonstrated that leisure-time physical activities or exercises are beneficial to prevent preterm birth^37^. The great difference in protection seemed to occur when women leading sedentary lifestyles were compared to those engaged in some type of physical activity^38^. A 2008 Brazilian study from Pelotas (a city in the south of Brazil) investigated the relationship between physical activity at leisure time (appropriate for each gestational trimester) and preterm birth. Leisure-time physical activity during the three trimesters of pregnancy was shown to offer protection against preterm birth (PR 0.55). Physical activity in the third trimester was also beneficial (PR = 0.50)^39^.

Favorable psychological or economic conditions determined by women entering the workforce and/or job satisfaction probably occur. These conditions may contribute to the release of substances that reduce the cascade of pathophysiological events triggering preterm labor in women genetically predisposed to a specific inflammatory response^40,41^. This hypothesis was not explored either in this study or in the literature and merits attention in the future. The wellbeing of an active pregnant woman at work, similar to the results observed when she exercises^25^, may contribute to better outcomes.

In addition to work characteristics, a higher BMI at the beginning of pregnancy and weight gain provided protection against sPTB^42^. There is conflicting evidence in the literature regarding the risk association between obesity and spontaneous preterm birth. A longer length of pregnancy and lower progression of labor have been described as resulting from abnormal myometrial contractility due to a decreased response of oxytocin in obese women^43–45^. On the other hand, pro-inflammatory status related to obesity has also been associated with a higher risk for sPTB and preterm premature rupture of membranes^46,47^. Nevertheless, weight gain did not explain the initial BMI and we used the total weight gain and not the weekly rate. Total weight gain does not control for pregnancy length. A secondary analysis focusing on the role of BMI and weight gain in the risk for sPTB was conducted for the EMIP study and gave a satisfactory explanation of these methodological details accordingly^48^.

Studies showing that anemia provided protection against sPTB were lacking. On the contrary, vast evidence has shown that anemia is a risk factor for sPTB^47^. We were unable to confirm whether changes had occurred during pregnancy, including the correction of anemia, instructions to reduce work activities and any other specific recommendation for this particular group of women.

The risk factors found in this study, such as previous preterm deliveries, polyhydramnios and vaginal bleeding, are in agreement with previous publications^47,49^. The study confirms classical risk factors, but draws attention to the protective role of maternal exertion, during paid work or while doing chores at home. Furthermore, some characteristics of occupational activities that considered risk factors for preterm delivery in the past are now in need of revision. Specific studies into the characteristics of different occupational activities, including associated physical effort, level of stress, and other possible benefits of working during pregnancy, should be carried out. Furthermore, the potential effects of supervised physical activities (exercise), leisure time, and household physical activities, and potential interactions need to be clarified. Finally, a change in physical activities at work, home, or leisure time during pregnancy, responding to expected complications, leading to reverse causality need to be controlled. The response to these questions is essential to provide pregnant women with better guidance on the individual risk of preterm delivery.

## Conclusion

Exertion during paid work was shown to provide protection against spontaneous preterm birth. The same occurred with working until the 8^th^ to 9^th^ month of pregnancy and completing domestic chores unaided. Protection may be due to a reverse causality or the benefits of working. These factors remain to be evaluated in the literature, concerning a sense of well-being and a perception of quality of life. Alternatively, it was probable that physical activity was moderately intense at the most, which is a factor associated with a better gestational prognosis in the literature.

## Supplementary information

Supplementary information.
