# Supplementary material for: Maternal Work and Spontaneous Preterm Birth: A Multicenter Observational Study in Brazil
Source: Sci Rep. 2020 Jun 16;10:9684. doi: 10.1038/s41598-020-66231-2 (PMC7297738; doi:10.1038/s41598-020-66231-2)
Supplement: Supplementary file 1 — Supplementary information. [file 41598_2020_66231_MOESM1_ESM.docx]

**Maternal Work and Spontaneous Preterm Birth: A Multicenter Observational Study in Brazil**

Mariana Buen, PE, MSc^1^; Eliana Amaral, MD, PhD^1^; Renato T Souza, MD, PhD^1^; Renato Passini Junior, MD, PhD^1^; Giuliane J Lajos, MD, PhD^1^; Ricardo P. Tedesco, MD, PhD^2^; Marcelo L. Nomura, MD, PhD^1^; Tábata Z. Dias, MD^1^; Patrícia M. Rehder, MD, PhD^1^; Maria Helena Sousa, Stat, PhD^2^, José Guilherme Cecatti, MD, PhD^1^; for the Brazilian Multicentre Study on Preterm Birth Study Group

**Affiliations**

^1^Department of Obstetrics & Gynecology, University of Campinas (Unicamp), School of Medicine, Brazil;

^2^ Jundiai School of Medicine, Jundiai, SP, Brazil

**S1. Method for estimating gestational age in the EMIP study**

| **Method** | **sPTB**  **N(%)** | **Term birth**  **N(%)** | **p-value***  **0.383** |
| --- | --- | --- | --- |
| LMP | 505 (39.5) | 428 (37.7) |  |
| US | 541 (42.3) | 559 (49.3) |  |
| New Ballard | 234 (18.2%) | 147 (13.0) |  |
| Total | 1,134 (100) | 1,280 (100) |  |

*qui-squared test
